# Oscillating viscous flow past a streamwise linear array of circular cylinders

**DOI:** 10.1017/jfm.2023.178

**Published:** 2023-03-24

**Authors:** J. Alaminos-Quesada, J.J. Lawrence, W. Coenen, A.L. Sánchez

**Affiliations:** 1 Department of Mechanical and Aerospace Engineering, University of California San Diego, La Jolla, CA 92093, USA; 2 Grupo de Mecánica de Fluidos, Departamento de Ingeniería Térmica y de Fluidos, Universidad Carlos III de Madrid, 28911 Leganés, Madrid, Spain

**Keywords:** general fluid mechanics

## Abstract

This paper addresses the viscous flow developing about an array of equally spaced identical circular cylinders aligned with an incompressible fluid stream whose velocity oscillates periodically in time. The focus of the analysis is on harmonically oscillating flows with stroke lengths that are comparable to or smaller than the cylinder radius, such that the flow remains two-dimensional, time-periodic and symmetric with respect to the centreline. Specific consideration is given to the limit of asymptotically small stroke lengths, in which the flow is harmonic at leading order, with the first-order corrections exhibiting a steady-streaming component, which is computed here along with the accompanying Stokes drift. As in the familiar case of oscillating flow over a single cylinder, for small stroke lengths, the associated time-averaged Lagrangian velocity field, given by the sum of the steady-streaming and Stokes-drift components, displays recirculating vortices, which are quantified for different values of the two relevant controlling parameters, namely, the Womersley number and the ratio of the inter-cylinder distance to the cylinder radius. Comparisons with results of direct numerical simulations indicate that the description of the Lagrangian mean flow for infinitesimally small values of the stroke length remains reasonably accurate even when the stroke length is comparable to the cylinder radius. The numerical integrations are also used to quantify the streamwise flow rate induced by the presence of the cylinder array in cases where the periodic surrounding motion is driven by an anharmonic pressure gradient, a problem of interest in connection with the oscillating flow of cerebrospinal fluid around the nerve roots located along the spinal canal.

## 1. Introduction

The interaction of an oscillating stream with velocity $U_{\infty }\text{ cos}\left(\omega t^{\prime }\right)$ with a fixed solid body is known to result in a time-averaged steady-streaming motion (Riley 2001). The solution that appears depends on the velocity amplitude $U_{\infty }$, the typical size of the object a, the oscillation frequency ω and the kinematic viscosity of the fluid v, which can be used to define two controlling parameters, namely, a dimensionless stroke length

(1.1) $$\varepsilon =\frac{U_{\infty }/\omega }{a}$$

and a Womersley number

(1.2) $$M={\left(\frac{a^{2}\omega }{v}\right)}^{1/2},$$

related to the Reynolds number according to $\text{Re}=U_{\infty }a/\nu =\varepsilon M^{2}$. For small values of ε, the problem is amenable to a theoretical description, wherein the velocity components are expressed as an asymptotic expansion involving powers of ε. The leading-order terms, satisfying convection-free linear equations, are harmonic functions with zero time-averaged values, while the first-order corrections have a non-zero steady-streaming component (Riley 2001). The resulting motion involves fundamentally two different time scales, a short time scale $\omega^{-1}$, associated with the fast oscillations of small amplitude εa occurring at leading order, and a slow-drift long-time scale $a/\left(\varepsilon U_{\infty }\right)=\varepsilon^{-2}\omega^{-1}$, required for the steady-streaming velocity, of order $\sim \varepsilon U_{\infty }$, to produce displacements of order a.

For the canonical case of two-dimensional flow over a circular cylinder of radius a, an analytical description of the Eulerian velocity for ε≪1 was found by Holtsmark *et al*. (1954), with expressions given for the leading-order harmonic velocity and for the first-order velocity corrections (errors in the latter were subsequently corrected by Chong *et al*. (2013)). In the distinguished regime M~1 considered by Holtsmark *et al*. (1954), the magnitude of the resulting steady-streaming velocity is comparable to that of the so-called Stokes drift, as demonstrated by Raney, Corelli & Westervelt (1954), so that the description of the drift experienced by the fluid particles requires consideration of both effects. Owing to the symmetry of the problem, the resulting Lagrangian mean motion displays identical recirculatory patterns in all four quadrants. For M below a critical value, a single vortex appears in each quadrant, with the motion directed towards the cylinder along the oscillation axis. A second vortex, external to the original vortex, appears for sufficiently large values of M, an interesting feature of the analytical solution verified by accompanying experiments (Holtsmark *et al*. 1954). This outer vortex increases in strength as M increases, while the inner vortex shrinks in size, such that, for M≫1, the latter is confined to a thin near-surface Stokes layer. The theoretical description of the flow arising for ε≪1 and M≫1 uses the distinguished limit of order-unity streaming Reynolds numbers $Re_{s}=\varepsilon^{2}M^{2}\sim 1$ (Stuart 1963, 1966; Riley 1965, 1967). The steady-streaming flow is seen to be determined in that case from the full equations of motion for steady viscous flow at Reynolds number $Re_{s}$, with limiting solutions arising for $Re_{s}\ll 1$ and $Re_{s}\gg 1$ (Riley 1967).

While the oscillating flow for ε≪1 remains periodic and symmetric about the oscillation axis, the solution encountered when ε takes values that are not sufficiently small is known to be more complicated. The periodic viscous flow becomes unstable to axially periodic vortices above a critical value of ε that depends on M (Hall 1984), leading to an asymmetrical flow with vortex shedding. (Note that most of the literature investigating velocity amplitudes that are not small use the oscillation period 2π/ω and the cylinder diameter 2a as characteristic scales of time and length, so that the Keulegan–Carpenter number $KC=U_{\infty }\left(2\pi /\omega \right)/\left(2a\right)=\pi \varepsilon$ and the Stokes number $\beta ={\left(2a\right)}^{2}/\left(v2\pi /\omega \right)=\left(2/\pi \right)M^{2}$ replace ε and M in the parametric description of the solution.) This symmetry breaking is apparent in the experiments of Tatsuno & Bearman (1990). The emerging flow exhibits a three-dimensional structure (Honji 1981), with turbulent motion arising as the Reynolds number $Re=\varepsilon M^{2}$ exceeds a critical value (Tatsuno & Bearman 1990).

Although the circular cylinder has attracted considerable attention, analyses of oscillating flows involving obstacles of differing shape are also available, including non-circular cylinders (Bearman *et al*. 1985), spheres (Lane 1955; Riley 1966), cylindrical posts confined between parallel walls (Rallabandi *et al*. 2015), three-dimensional multi-curvature bodies (Bhosale *et al*. 2022; Chan *et al*. 2022), cylinder pairs with either equal (Williamson 1985; Coenen & Riley 2008; Chong *et al*. 2016; Coenen 2016) or unequal radii (Coenen 2013) and three-cylinder arrays in different arrangements (Chong *et al*. 2016). Multiple circular cylinders arranged in periodic, regular lattices have also been investigated, including square arrays of identical cylinders (House, Lieu & Schwartz 2014) and square arrays involving cylinders with two different radii (Bhosale, Parthasarathy & Gazzola 2020). A linear array of equally spaced identical circular cylinders performing harmonic oscillations in the transverse direction in a fluid that is otherwise at rest was considered in the numerical and experimental work of Yan, Ingham & Morton (1993, 1994). The resulting steady streaming, identical to that found when a fixed cylinder array is placed perpendicular to a harmonically oscillating stream, was evaluated in the limit ε≪1 with $Re_{s}\sim 1$.

**Figure 1.**
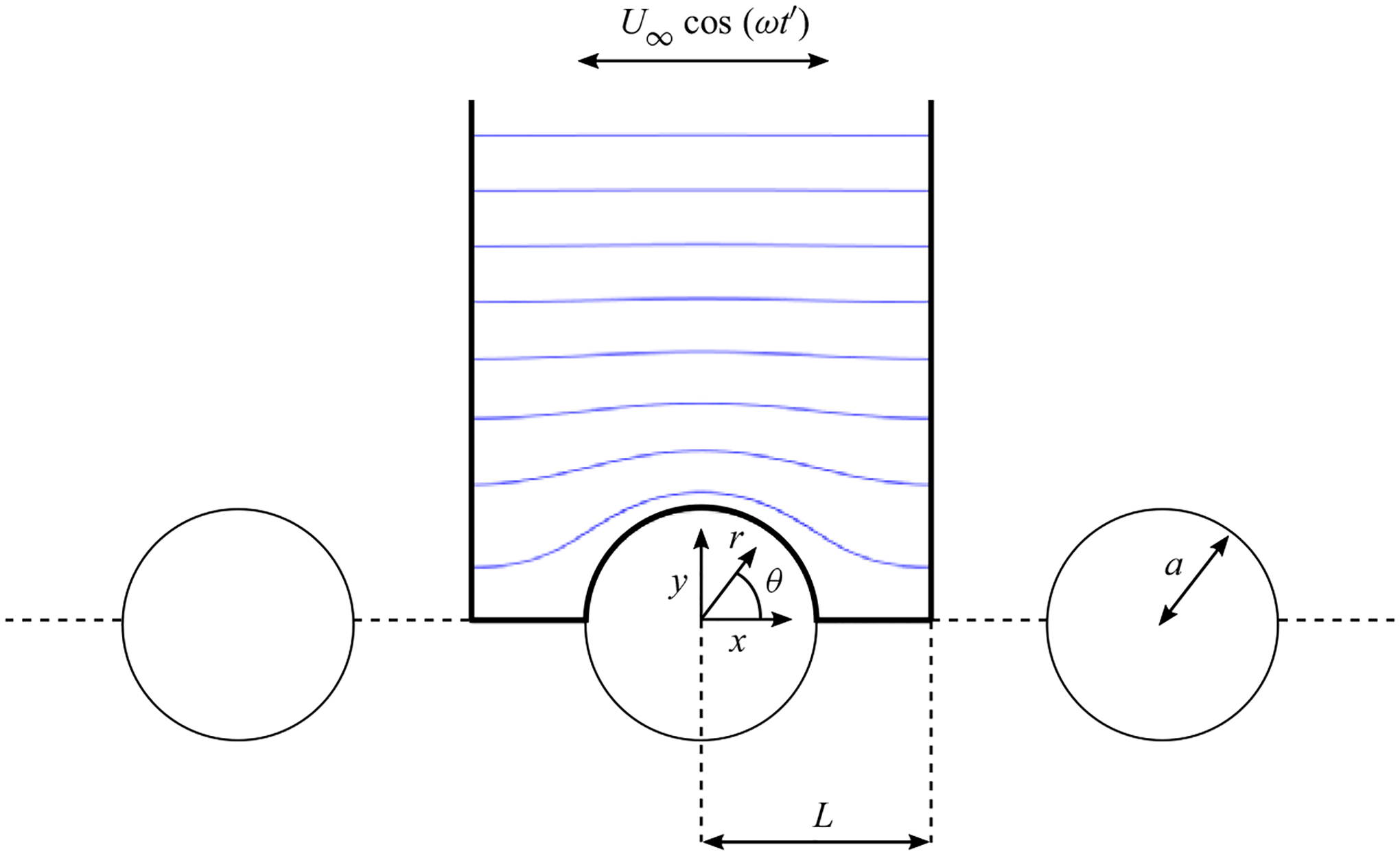
Schematic illustration of the cylinder array for ℓ=L/a=2, including the streamlines corresponding to the potential-flow solution.

To the best of our knowledge, situations in which the obstacle array is aligned with the oscillating stream have not yet been considered. As a first step to elucidate the associated dynamics, the present study considers the canonical configuration schematically represented in figure 1, involving a row of uniformly spaced circular cylinders aligned with the oscillating stream. This flow configuration can be seen as a variant of the problem considered by Yan *et al*. (1993. 1994), in which the cylinder array was oscillating perpendicular to the array axis. Attention is directed to configurations with Womersley numbers M≳1 and values of the stroke length that are either ε≪1 or ε~1. This parametric range corresponds to a regime of moderate Reynolds numbers $Re=U_{\infty }a/v=\varepsilon M^{2}$ where the solution is free from asymmetric vortex shedding (Tatsuno & Bearman 1990; Yan *et al*. 1993, 1994), so that the associated two-dimensional time-periodic flow displays symmetry with respect to the oscillation axis.

The analysis of steady streaming in the array configuration analysed here is relevant in connection with microscale fluid devices, including applications targeting particle manipulation (Lutz, Chen & Schwartz 2005, 2006; Chong *et al*. 2013; Huang *et al*. 2013; House *et al*. 2014). Oscillating flows featuring interactions with streamwise obstacle arrays are found in other problems, an example being the flow of cerebrospinal fluid (CSF) in the spinal subarachnoid space, a slender annular canal that surrounds the spinal cord. The pulsating motion of CSF, driven by the pressure oscillations induced by the cardiac and respiratory cycles (Linninger *et al*. 2016), exhibits velocities that vary along the canal. For example, for the cardiac-driven flow, the peak velocity decays from values of the order of a few centimetres per second in the cervical region to values of the order of a few millimetres per second in the lumbar region (Coenen *et al*. 2019, figure 2). This pulsatile motion is affected by the presence of nerve roots, which has been found to enhance steady streaming (Khani *et al*. 2018) and local mixing (Pahlavian *et al*. 2014), thereby promoting the transport of solutes along the canal (Stockman 2006, 2007). These nerve roots, which branch off the spinal cord to deliver nerve signals to the rest of the body (Sass *et al*. 2017), are arranged in quasi-periodic rows aligned along the canal, with the axial distance between subsequent nerve roots determined by the inter-vertebra spacing. Each nerve root consists of multiple rootlets arranged in bundles, forming a structure whose streamwise length varies from about 1 mm near the external dura membrane, where the nerve root is more round, to about 1 cm at the root base near the spinal cord (Mendez *et al*. 2021, figures 1 and 2). The resulting pulsatile flow about the nerve root is characterized by moderately large values of the Womersley number in the range 6<M<15, as can be seen by evaluating (1.2) with the cardiac angular frequency $\omega =2\pi {\text{s}}^{-1}$ and the CSF kinematic viscosity $v=0.7{\text{ mm}}^{2}{\text{ s}}^{-1}$ for an obstacle of size a=2−5 mm. The value of the dimensionless stroke length ε evaluated from (1.1) is of order unity in the cervical region (e.g. ε≃1.6 for $U_{\infty }=2{\text{ cm s}}^{-1}$ and a=2 mm) and small in the lumbar region (e.g. ε≃0.16 for $U_{\infty }=2{\text{ mm s}}^{-1}$ and a=2 mm).

The rest of the paper is organized as follows. After formulating the problem in § 2, we address in § 3 the limit of small stroke lengths ε≪1. Following the standard asymptotic treatment of steady-streaming problems (Riley 2001), the solution uses expansions for the different variables in powers of ε, leading to a hierarchy of problems that can be solved sequentially, with the steady-streaming velocity obtained by time-averaging the first-order velocity corrections. Unlike the case of a single cylinder, for which closed-form analytic solutions are available (Holtsmark *et al*. 1954; Chong *et al*. 2013), for the cylinder array numerical computation is needed to solve the problems that emerge at the different orders. For the case M~1 considered here, it will be shown that the resulting steady-streaming velocity is comparable to the Stokes drift, in agreement with previous results (Raney *et al*. 1954; Chong *et al*. 2013). Direct numerical simulations (DNS) will be used in § 4 to investigate the mean Lagrangian motion arising for ε~1 and to test the range of validity of the ε≪1 description. Besides harmonically oscillating streams, resulting in steady-streaming patterns with closed recirculating streamlines, similar to those found earlier (Holtsmark *et al*. 1954), consideration will be given in § 5 to configurations with periodic anharmonic flow, that being the case of the oscillating motion in the spinal canal. An important related question addressed below is whether the interactions of an obstacle row with an anharmonic oscillating stream of zero mean velocity may produce a non-zero streamwise net flow rate, which might explain previous observations regarding transport-rate enhancement along the canal (Stockman 2006, 2007). Finally, concluding remarks are given in §6.

## 2. Formulation

Let us consider the flow configuration depicted in figure 1, emerging when an incompressible fluid stream with harmonic velocity $U_{\infty }\text{cos}\left(\omega t^{\prime }\right)$ flows past an infinite array of equally spaced identical cylinders aligned with the unperturbed flow. The semi-distance L between the centres of contiguous cylinders is assumed to be comparable to the cylinders radius a, their ratio defining the geometrical parameter ℓ=L/a⩾1. As previously anticipated, the two controlling flow parameters are the dimensionless stroke length ε, defined in (1.1), and the Womersley number M, defined in (1.2). DNS corresponding to order-unity values of the three parameters ℓ,M and ε are to be presented below along with results corresponding to the small-stroke-length asymptotic limit ε≪1, when the velocity displays a harmonic temporal dependence at leading order, while the first-order corrections, of order $\varepsilon U_{\infty }$, contain a steady contribution.

The problem is scaled with use of $a,\omega^{-1},U_{\infty }$ and $\rho \omega aU_{\infty }$ as characteristic values of length, time, velocity and spatial pressure difference, with ρ denoting the fluid density. Correspondingly, the unperturbed flow velocity of the external oscillating stream becomes $u_{\infty }=\text{cos }t$ with $t=\omega t^{\prime }$. Since the resulting velocity v is periodic in the streamwise direction, the solution can be described by considering the flow about an individual cylinder, with the origin of the coordinate system placed at the cylinder centre. The description employs Cartesian coordinates x=(x,y) and Cartesian velocity components v=(u,v), with x aligned in the direction of the unperturbed flow and $r={\left(x^{2}+y^{2}\right)}^{1/2}$ denoting the distance to the cylinder centre, as indicated in figure 1. Since, in the regime ε≲1 and M~1 investigated below, the flow can be anticipated to remain symmetric about the y=0 plane, in the computations it suffices to consider the integration domain extending for $x^{2}+y^{2}>1$ with y>0 and −ℓ<x<ℓ. The velocity must satisfy the continuity and momentum equations

(2.1) ∇⋅v=0

(2.2) $$\frac{\partial v}{\partial t}+\varepsilon v\cdot \nabla v=-\nabla p+\frac{1}{M^{2}}\nabla^{2}v,$$

subject to the non-slip condition

(2.3) v=0  at r=1,

the far-field condition

(2.4) v=(cos t,0) as y→∞  for −ℓ⩽x⩽ℓ,

the centreline symmetry condition

(2.5) $$\frac{\partial u}{\partial y}=v=0\text{ at }y=0\text{ for }1⩽\left|x\right|⩽\ell$$

and the condition of 2ℓ spatial periodicity in the x direction. The free-stream velocity condition (2.4) is consistent with a far-field pressure distribution approaching p=x sin t as y→∞ The above problem was integrated numerically using the immersed boundary method with the projection approach given by Taira & Colonius (2007) in a Cartesian non-uniform staggered mesh extending up to y=30. The value of the associated grid spacing Δ, smaller near the cylinder surface, was reduced for increasing values of the Womersley number as needed to resolve the associated near-wall Stokes layer with sufficient accuracy, yielding, for instance, Δ=0.04 for M=1 and Δ=0.01 for M=16. The spatial width of the cylinder nodes employed in the implementation of the immersed boundary method was selected to be equal to the smallest spacing of the Cartesian mesh. The time step δt was correspondingly adjusted to give a Courant number δt/Δ below 0.25. Following standard practice (see e.g. Alaminos-Quesada 2021), the implementation of the far-field condition (2.4) was facilitated in the simulations by rewriting the problem in terms of the axial velocity perturbation $u^{\prime }=u-\text{cos }t$, which satisfies $u^{\prime }=-\text{cos }t$ at r=1 and $u^{\prime }\to 0$ as y→∞ along with the symmetry and periodicity conditions stated above. As explained in Appendix A, the numerical method was validated through comparisons with previously reported results corresponding to a single cylinder.

## 3. The limit of small stroke lengths

Following standard practice, the flow description in the limit ε≪1 utilizes expansions for the different flow variables in powers of ε, i.e. $v=v_{0}+\varepsilon v_{1}+\cdots$ and $p=p_{0}+\varepsilon p_{1}+\cdots$. As seen below, the leading-order solution has a zero time average, i.e. ${\langle v\rangle }_{0}=0$, with $\langle \cdot \rangle =\left(1/2\pi \right)\int_{t}^{t+2\pi }\cdot \text{d}t$, whereas the first-order correction $v_{1}$, accounting for the effects of convective acceleration, includes a non-zero steady-streaming component $v_{SS}=v_{1}$

### 3.1. Leading-order oscillatory flow

At leading order in the limit ε≪1, convective acceleration does not enter in the momentum balance equation (2.2). The resulting linear problem can be conveniently solved by introducing $v_{0}=\text{Re}\left({\text{e}}^{\text{i}t}V\right)$ and $p_{0}=\text{Re}\left({\text{e}}^{\text{i}t}P\right)$ with V(x,y)=(U,V) and P(x,y) representing complex functions satisfying

(3.1a,b) $$\nabla \cdot V=0,\text{ i}V=-\nabla P+\frac{1}{M^{2}}\nabla^{2}V,$$

with boundary conditions

(3.2) $$\begin{array}{lll} V=0 & \text{ at }r=1, & \\ V=\left(1,0\right) & \text{ as }y\to \infty & \text{ for }-\ell ⩽x⩽\ell ,\\ \partial U/\partial y=V=0 & \text{ at }y=0 & \text{ for }1⩽\left|x\right|⩽\ell , \end{array}\}$$

as follows from (2.1)–(2.5), along with the condition of 2ℓ spatial periodicity in the x direction.

Except for the limiting case ℓ≫1, which reduces to that of flow over a single cylinder (Holtsmark *et al*. 1954; Chong *et al*. 2013), no analytic solution is available, and the above problem must be solved numerically. To that aim, (3.1a,b) were written in weak form and implemented in the finite element solver COMSOL Multiphysics. Solutions were computed on an unstructured triangular mesh that extended laterally to y=30. Mesh elements were clustered near the cylinder surface, the typical element size ranging from 0.01 at that surface to 0.2 near the far-field boundary. It was checked that further increases in lateral domain extension as well as in mesh refinement did not alter the results.

For a general value of M, the resulting complex velocity V(x,y) has real and imaginary parts. Note, however, that, in the inviscid limit M≫1, the solution contains an imaginary part only in the thin Stokes layer of thickness 1/M that develops on the cylinder surface, outside of which the flow is irrotational, such that V(x,y)=∇Φ. The associated velocity potential Φ, a real function, satisfies $\nabla^{2}\Phi =0$ subject to ideal-flow boundary conditions stemming from (3.2), including, for instance, the no-penetration condition ∂Φ/∂r=0 at r=1. The problem was considered recently by Crowdy (2016), who provided a quasi-analytical solution for the corresponding complex potential. For illustrative purposes, the streamlines of the potential flow corresponding to the specific case ℓ=2 are included in the schematic of figure 1.

### 3.2. Steady streaming

The steady-streaming velocity ${\text{v}}_{SS}=\langle {\text{v}}_{1}\rangle =\left(u_{SS},v_{SS}\right)$ is determined from the problem that arises at the following order. Collecting terms of order ε in (2.1) and (2.2) and taking the time average leads to

(3.3a,b) $$\nabla \cdot v_{SS}=0,\;\frac{1}{2}\text{Re}\left(V\cdot \nabla V^{\text{*}}\right)=-\nabla \langle p_{1}\rangle +\frac{1}{M^{2}}\nabla^{2}v_{SS},$$

after writing $\langle v_{0}\cdot \nabla v_{0}\rangle =\frac{1}{2}\text{Re}\left(V\cdot \nabla V^{\text{*}}\right)$, which follows from the identity

(3.4) $$\langle \text{Re}\left({\text{e}}^{\text{i}t}A\right)\text{Re}\left({\text{e}}^{\text{i}t}B\right)\rangle =\text{Re}\left(AB^{\text{*}}\right)/2,$$

applying to any generic time-independent complex functions A and B, with the asterisk * denoting complex conjugates. The resulting recirculating cells, symmetric about the x=0 plane, can be correspondingly obtained by integrating (3.3a,b) in the first quadrant subject to the boundary conditions

(3.5) $$\begin{array}{lll} v_{SS}=0 & \text{ at }r=1, & \\ v_{SS}\to 0 & \text{ as }y\to \infty & \text{ for }0⩽x⩽\ell ,\\ \partial u_{SS}/\partial y=v_{SS}=0 & \text{ at }y=0 & \text{ for }1⩽x⩽\ell , \end{array}\}$$

consistent with (2.3)–(2.5), and the condition of 2ℓ spatial periodicity in the x direction. At this order, the steady-streaming pressure $\langle p_{1}\rangle$ vanishes in the far field, as is consistent with the velocity condition $v_{SS}\to 0$ as y→∞.

**Figure 2.**
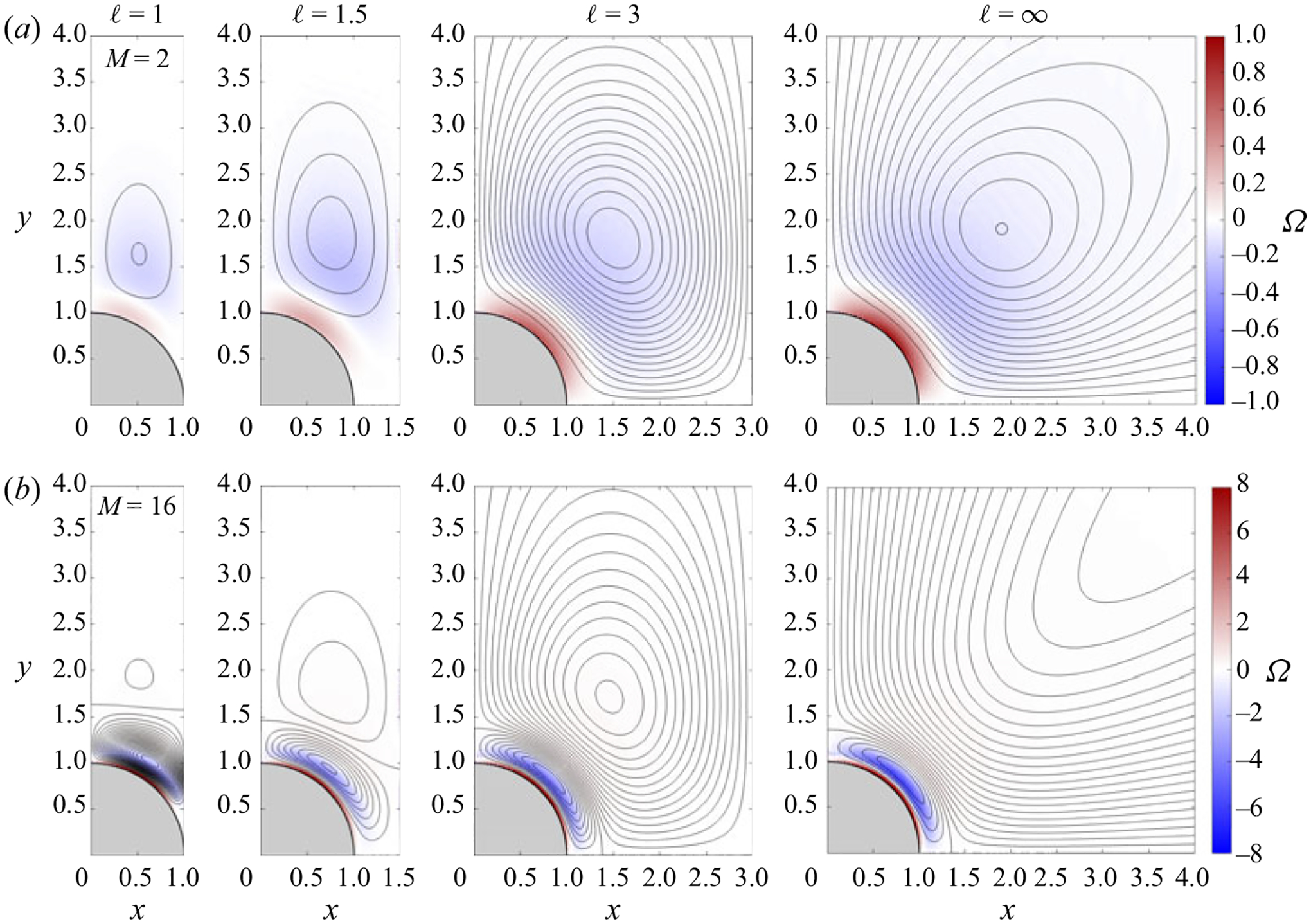
Streamlines and colour contours of vorticity Ω corresponding to the steady-streaming motion with different inter-cylinder distance ℓ for M=2 (*a*) and M=16 (*b*). Streamlines are represented using a constant spacing δψ, with δψ=0.002 for ℓ=1, δψ=0.005 for ℓ=1.5 and 3, and δψ=0.01 for ℓ=∞. Corresponding vorticity levels are indicated in the colour bar on the right.

Equations (3.3a,b) were integrated using the numerical method employed earlier for the leading-order problem. Representative results are shown in figure 2 for four values of the inter-cylinder spacing ℓ, including as extreme cases the configuration with touching cylinders (ℓ=1) and the familiar single-cylinder case, recovered in the present array configuration when ℓ=∞. Because of the condition of flow periodicity and the symmetry of the cylinder array, the vertical lines x=0,1⩽y<∞ and x=ℓ,0⩽y<∞ are streamlines of the steady-streaming flow. Only the first quadrant is shown in figure 2, since the flow structure is identical in all four quadrants. Streamlines are plotted using a fixed increment δψ of the streamfunction $\psi_{SS}$ computed from $\partial \psi_{SS}/\partial y=u_{SS}$ and $\partial \psi_{SS}/\partial x=-v_{SS}$, with $\psi_{SS}=0$ on the domain boundary. The spacing is δψ=0.005 for ℓ=1.5 and ℓ=3.0, with a smaller spacing δψ=0.002 used for ℓ=1, as needed to represent the associated weak motion, and a larger spacing δψ=0.01 for ℓ=∞, in accordance with the associated vigorous motion. In addition to streamlines, colour contours are used to represent the vorticity Ω=∂v/∂x−∂u/∂y, with the level indicated in the colour bar on the far right.

As seen in figure 2, the streaming structure arising for finite values of ℓ is qualitatively similar to that of a single cylinder (Holtsmark *et al*. 1954). For M=2 the flow displays one vortex in each quadrant, with the clockwise circulation (negative vorticity) exhibited by the vortex in the first quadrant corresponding to fluid approaching the cylinder along the oscillation axis y=0. This vortex is known to progressively approach the cylinder wall on increasing M (Holtsmark *et al*. 1954) and, for the case M=16 shown in figure 2(b), is seen to be embedded in the high-vorticity Stokes layer that develops near the cylinder surface. A second vortex with opposite circulation, clearly visible in the results for M=16, appears outside in configurations with M exceeding a critical value $M_{c}$. For the case of a single cylinder, the value $M_{c}≃6.08$ can be determined from the exact solution (Holtsmark *et al*. 1954) as the value of M for which the streamfunction $\psi_{SS}$ vanishes in the far field. From our numerical computations, it was seen that the value of $M_{c}$ is somewhat larger for the cylinder array (e.g. $M_{c}≃7$ for ℓ=2).

The presence of the neighbouring cylinders has a noticeable effect on the shape of the resulting vortices, as can be seen by comparing the results for ℓ=(1,1.5,3) with the canonical case of a single cylinder (ℓ=∞) shown in the last column of figure 2. For M=2 the core of the vortex, which for ℓ=∞ is located along the π/4 ray, is displaced towards the vertical axis x=0 on decreasing the inter-cylinder spacing, producing vortices that are much more slender, with the case ℓ=1 displaying the largest distortion. For M=16, the outer vortex, which for the single cylinder exhibits open streamlines with no vortex core, displays for ℓ≠∞ a well-defined core surrounded by closed streamlines. This qualitative change, also observed in the flow about an oscillating cylinder when enclosed by a concentric cylindrical surface (Holtsmark *et al*. 1954), is attributable to the effect of confinement, which also produces a drastic reduction in the magnitude of the streaming motion. The extent of the reduction can be quantified by comparing the peak value of the streamfunction, given by $\psi_{\mathrm{SS},\text{peak }}=-0.1602$ for M=2 and $\psi_{\mathrm{SS},\text{peak}}=\left(-0.0493/0.243\right)$ (inner/outer vortex) for M=16 in the case of the isolated cylinder (ℓ=∞) and $\psi_{\mathrm{SS},\text{peak }}=-0.0041$ for M=2 and by $\psi_{\mathrm{SS},\text{peak}}=\left(-0.0438/0.0022\right)$ (inner/outer vortex) for M=16 in the case of an array of touching cylinders (ℓ=1).

### 3.3. Mean Eulerian velocity for finite stroke lengths

The steady-streaming velocity $v_{SS}=\langle v_{1}\rangle$ provides the leading-order description for the mean Eulerian velocity $\langle v\rangle =\varepsilon v_{SS}$ in the asymptotic limit ε≪1. In principle, the description can be improved by computing higher-order terms in the asymptotic expansion for $\langle v\rangle =\varepsilon \langle v_{1}\rangle +\varepsilon^{2}\langle v_{2}\rangle +\varepsilon^{3}\langle v_{3}\rangle +\cdots$. The development must begin by computing the unsteady component of the first-order velocity correction $v_{1}$, which can be shown to be of the form $v_{1}-\langle v_{1}\rangle =\text{Re}\left({\text{e}}^{2\text{it}}V_{1}\right)$, where $V_{1}\left(x,r\right)$ is a complex function, the expression of which was obtained by Chong *et al*. (2013) for the case of a single isolated cylinder. The equations that determine $\langle v_{2}\rangle$, analogous to (3.3a,b), with the convective term in the momentum equation replaced by $\langle v_{0}\cdot \nabla v_{1}\rangle +\langle v_{1}\cdot \nabla v_{0}\rangle$, are to be integrated with the homogeneous boundary conditions stated in (3.5), with $\langle v_{2}\rangle$ replacing $v_{SS}$. Since $v_{0}=\text{Re}\left({\text{e}}^{\text{i}t}V\right)$ and ${\langle v\rangle }_{1}={\langle v\rangle }_{1}+\text{Re}\left({\text{e}}^{2\text{i}t}V_{1}\right)$, it follows that $\langle v_{0}\cdot \nabla v_{1}\rangle +\langle v_{1}\cdot \nabla v_{0}\rangle =0$, with the consequence that integration of the steady-streaming problem that arises at order $\varepsilon^{2}$ yields $\langle v_{2}\rangle =0$. Therefore, the corrections to the mean Eulerian velocity would enter only at the following order, i.e. $\langle v\rangle =\varepsilon \langle v_{1}\rangle +\varepsilon^{3}\langle v_{3}\rangle +\cdots$, indicating that the leading-order expression $\langle v\rangle =\varepsilon v_{SS}=\varepsilon \langle v_{1}\rangle$ computed here contains small relative errors of order $\varepsilon^{2}$.

**Figure 3.**
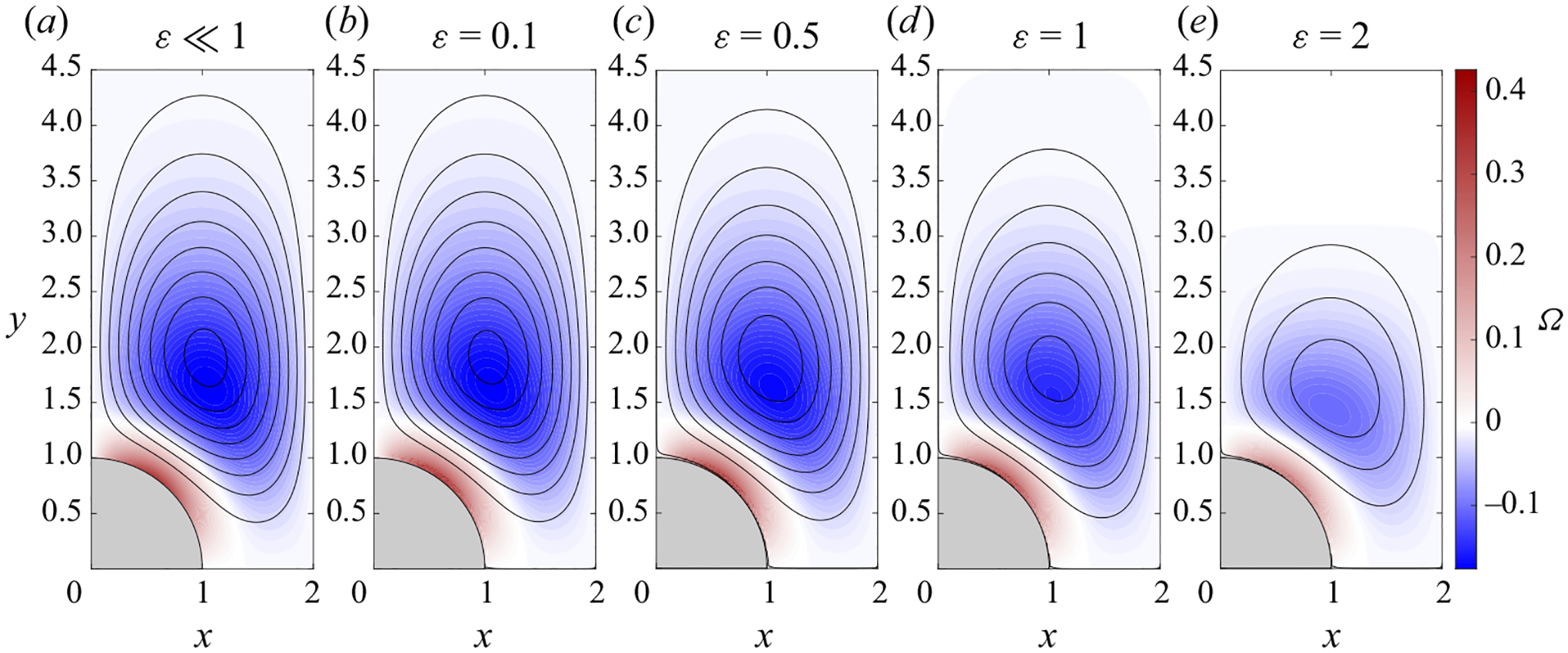
Streamlines and colour contours of vorticity Ω for ℓ=2 and M=2. Besides results corresponding to the steady-streaming velocity $v_{SS}$, shown in panel (*a*), results are given for the rescaled time-averaged Eulerian velocity 〈v〉/ε determined in the DNS computations for ε=(0.1, 0.5, 1.0, 2.0) in panels (*b*)–(*e*). Streamlines are represented using a constant spacing δψ=0.005. Corresponding vorticity levels are indicated in the colour bar on the right.

The accuracy of the asymptotic description $\langle v\rangle =\varepsilon v_{SS}$ was tested through comparisons with the mean Eulerian velocity $\langle v\rangle =\left(1/2\pi \right)\int_{t}^{t+2\pi }v\text{ d}t$ determined in direct integrations of the complete problem (2.1)–(2.5). Selected numerical results corresponding to ℓ=2 and M=2 are shown in figure 3(b–e) for ε=(0.1,0.5,1.0,2.0). Since the time-averaged velocity can be anticipated to be of order ε, as suggested by the asymptotic analysis for ε≪ 1, the rescaled velocity 〈v〉/ε is used in computing the streamlines and vorticity contours shown in figure 3. The results are to be compared with those of the steady-streaming velocity $v_{SS}$, shown in figure 3(a). Close agreement is found between the DNS results for ε=0.1 and the ε≪1 predictions, with the associated velocity fields being nearly identical, as seen in figure 3. A quantitative measure of the existing differences, of the order of 1% for ε=0.1, consistent with the relative errors of order $\varepsilon^{2}$ anticipated in the discussion of the preceding paragraph, is provided by the peak values of the corresponding streamfunctions at the vortex centre, given by $\psi_{SS}=-0.0419$ for ε≪1 and 〈ψ〉/ε=−0.0416 for ε=0.1. It is remarkable that, although larger differences are found as the oscillation amplitude becomes comparable to the cylinder radius, the ε≪1 description remains reasonably accurate even for ε=0.5, for which 〈ψ〉/ε=−0.0390 at the vortex centre. For completeness, a figure showing the spatial distribution of $\left|\psi_{SS}-\langle \psi \rangle /\varepsilon \right|$ is included in Appendix B.

### 3.4. Stokes drift

As pointed out by Raney *et al*. (1954) when addressing oscillating flow over a cylinder, the Lagrangian mean motion of the fluid particles comes partly from the Eulerian mean motion (i.e. $\langle v\rangle =\varepsilon v_{SS}$) and partly from the so-called Stokes drift (Stokes 1847), a purely kinematic effect arising in non-uniform oscillating flows. As a result, streamlines visualized in experiments by tracing the motion of dyed fluid do not coincide in general with those determined from the steady-streaming velocity (Raney *et al*. 1954; Larrieu, Hinch & Charru 2009; Chong *et al*. 2013). Since the velocity of the Lagrangian mean motion $v_{SS}+v_{SD}$, where

(3.6) $$v_{SD}=\langle \int \;v_{0}\text{ d}t\cdot \nabla v_{0}\rangle$$

represents the contribution of the Stokes drift, determines the convective transport of solutes, there is interest in quantifying numerically $v_{SD}$ for the cylinder array, thereby complementing the analytical results developed previously for the single cylinder (Holtsmark *et al*. 1954; Raney *et al*. 1954; Chong *et al*. 2013).

The expression (3.6) for the Stokes-drift velocity, which can be systematically derived using a two-time-scale analysis, as shown in Appendix C, can be written in the form

(3.7) $$v_{SD}=\frac{1}{2}\text{Im}\left(V\cdot \nabla V^{\text{*}}\right)$$

by using $v_{0}=\text{Re}\left({\text{e}}^{\text{i}t}V\right)$ along with the identity $\text{Re}\left({\text{ie}}^{\text{i}t}A\right)\text{Re}\left({\text{e}}^{\text{i}t}B\right)=-\text{Im}\left(AB^{\text{*}}\right)/2$. It is of interest that the real part of the complex function $\frac{1}{2}V\cdot \nabla V^{\text{*}}$ determines the steady streaming, as revealed by (3.3a,b), whereas its imaginary part is the Stokes-drift velocity (3.7). Note that, as mentioned before, for large values of M viscous forces are confined to a thin Stokes layer, outside of which the flow is potential and the function V is real, so that the associated Stokes drift can be expected to vanish for M≫1, as follows from (3.7).

### 3.5. Evaluation of the Lagrangian mean velocity

**Figure 4.**
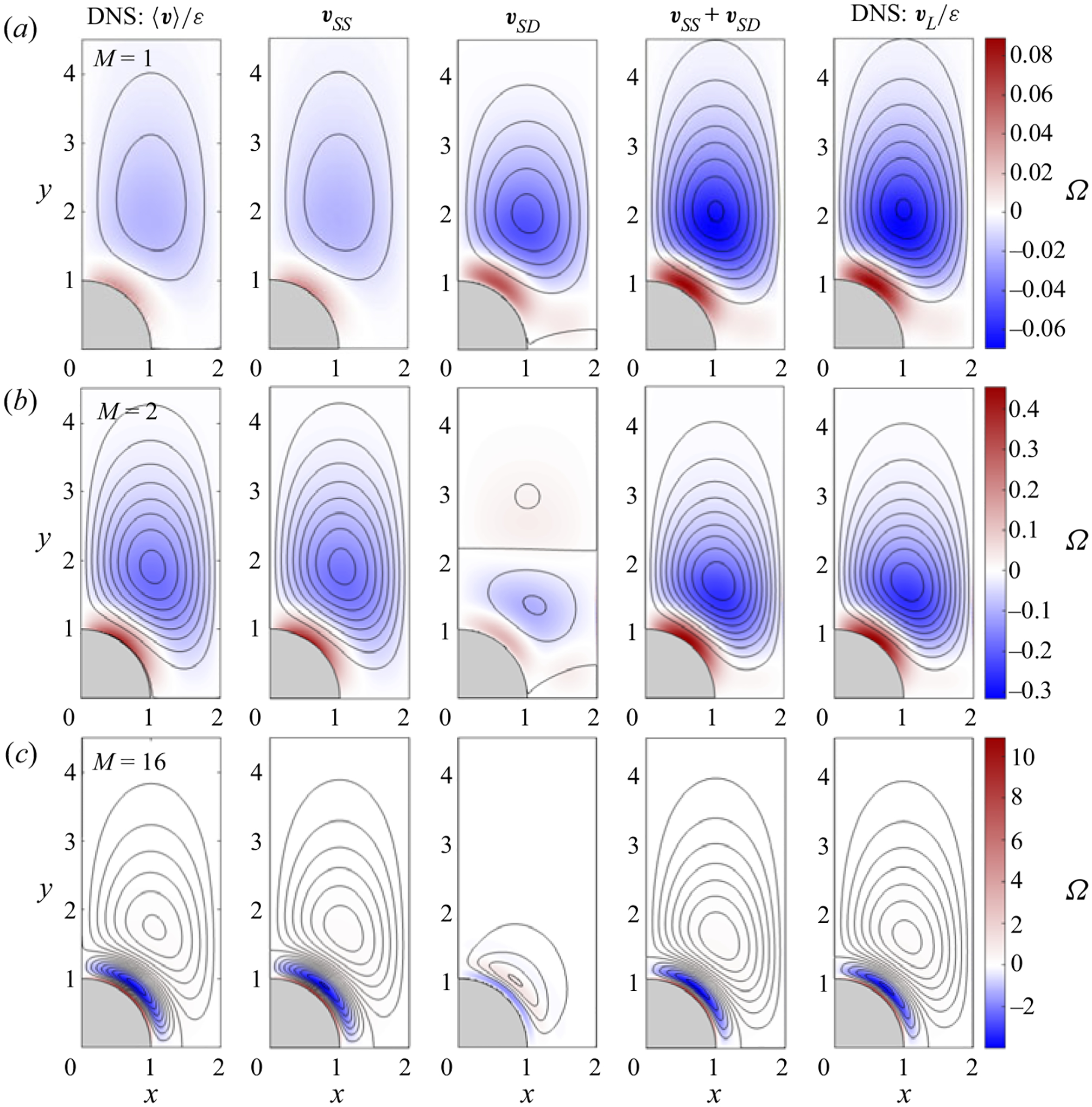
Streamlines and colour contours of vorticity Ω corresponding to the steady-streaming velocity $v_{SS}$, Stokes-drift velocity $v_{SD}$ and steady mean Lagrangian velocity $v_{L}=v_{SS}+v_{SD}$ for ℓ=2 and M=1 (*a*), M=2 (*b*) and M=16 (*c*). Corresponding DNS results for ε=0.1 are also shown, including the rescaled time-averaged Eulerian velocity field 〈v〉/ε (first column) and the rescaled Lagrangian velocity $v_{L}/\varepsilon$ (fifth column). For each value of M, streamlines are represented using a constant spacing δψ=0.002(M=1) and δψ=0.005 (M=2 and M=16), with the corresponding vorticity levels indicated in the colour bar on the right.

The expression (3.7) was used to evaluate the Stokes-drift velocity $v_{SD}$ for a cylinder array with ℓ=2, with associated streamlines and vorticity contours given in the middle column of figure 4. The first two columns of figure 4 show the corresponding steady-streaming velocity $v_{SS}$ (second column from the left) along with the rescaled time-averaged Eulerian velocity 〈v〉/ε determined in DNS computations with ε=0.1 (leftmost column), the two sets of results being nearly indistinguishable. Besides the two Womersley numbers M=2 and M=16 considered earlier in the computations of figure 2, figure 4 includes results for M=1, a case for which the Stokes drift is stronger than the steady-streaming motion. To facilitate comparisons, in plotting the streamlines for each value of M, the spacing of the Stokes-drift streamfunction $\psi_{SD}$ is that used for the corresponding steady-streaming plot.

**Figure 5.**
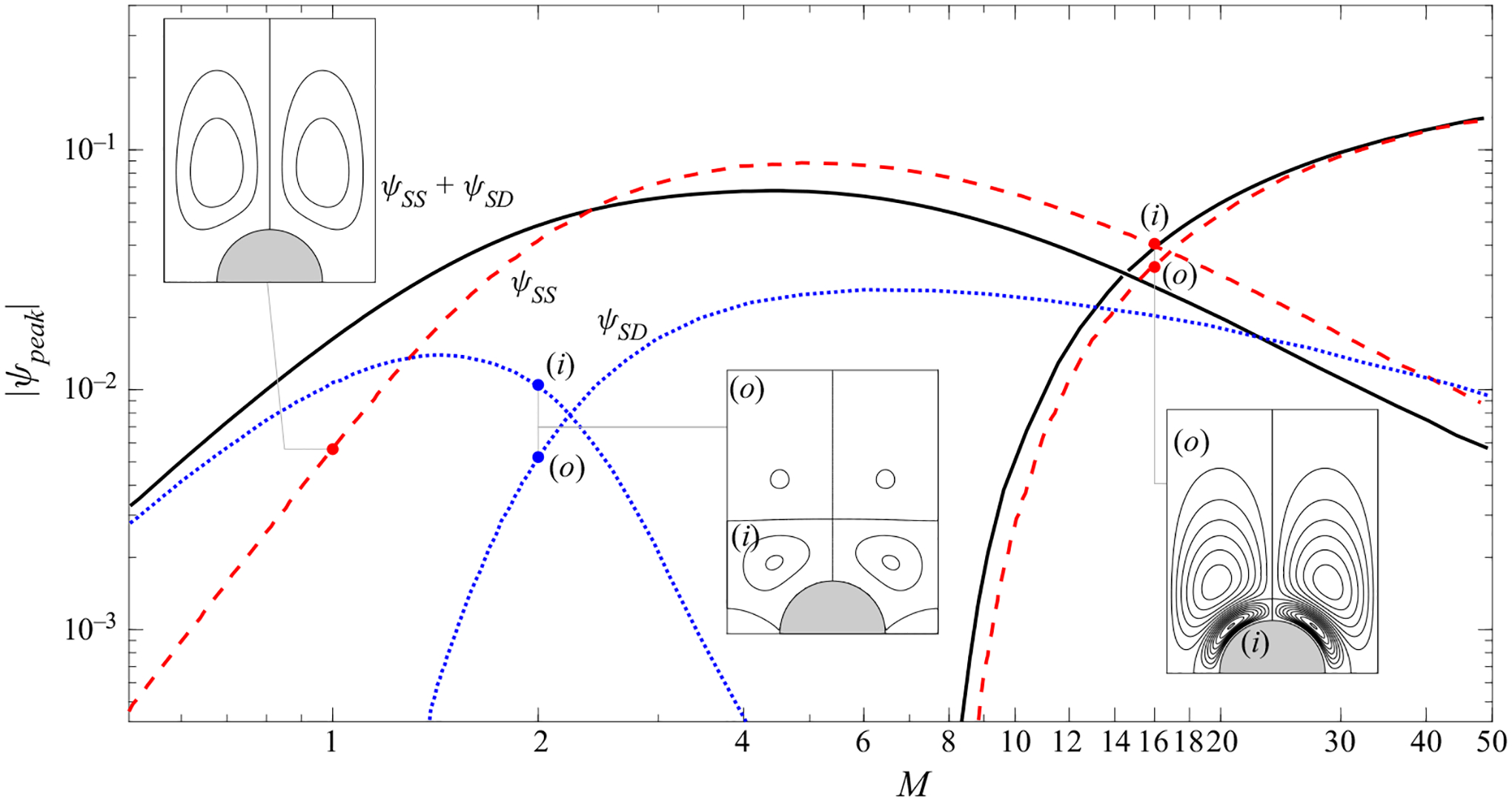
The variation with M of the magnitude $\left|\psi_{\text{peak}}\right|$ of the local peak values of the streamfunction $\psi_{SS}$ (dashed curves), $\psi_{SD}$ (dotted curves) and $\psi_{SS}+\psi_{SD}$ (solid curves) at the centre of the outer (o) and inner (i) vortices for the ℓ=2 configuration.

As can be seen, the Stokes-drift results for M=1 display a primary clockwise-rotating vortex occupying most of the quadrant, along with a much weaker counter-rotating vortex of negligibly small circulation near the oscillation axis y=0. For this value of M, this primary vortex is significantly stronger than the corresponding steady-streaming vortex. This can be verified by comparing the magnitude $\left|\psi_{\text{peak}}\right|$ of the peak values of the associated streamfunctions at the vortex centre. Since ψ is defined to be zero on the cylinder surface, the value of $\left|\psi_{\text{peak }}\right|$, whose variation with M is represented in figure 5, gives a measure of the volume flow rate driven by the recirculating vortex motion. As can be seen, for M=1 the peak value of $\psi_{SD}$ is significantly larger than that of $\psi_{SS}$, with the result that the Lagrangian velocity $v_{SS}+v_{SD}$ is largely determined by its Stokes-drift component, as reflected in the shape of the corresponding Lagrangian vortex, shown in the fourth column of figure 4(a).

The Stokes-drift motion develops an additional vortex, external to the primary vortex, when the Womersley number is increased to values exceeding a critical value (e.g. M≃1.5 for ℓ=2). As seen in the plots of peak streamfunction in figure 5, this external Stokes-drift vortex, clearly visible in figure 4(b), increases in strength for increasing M to prevail over the inner vortex for M≳2.5. Figure 5 also reveals that, for the cases M=2 and M=16 of figures 4(b) and 4(c), the Stokes drift is significantly weaker than the steady streaming, so that the Lagrangian motion is largely determined by the latter.

Figure 5 also shows the peak value of the streamfunction $\psi_{SS}+\psi_{SD}$ associated with the Lagrangian motion. Regarding the resulting curve, it is of interest that, since the inner and outer vortices have opposite circulation, leading to peak values of the streamfunction with different sign, there is an intermediate range of values of M for which the strength of the Lagrangian vortex is smaller than that of the steady-streaming vortex. The comparison of the different curves in figure 5 reveals that the Stokes drift prevails for sufficiently small values of the Womersley number M≪1, for which $\psi_{SS}\ll \psi_{SD}$, whereas in the opposite limit M≫1 the Stokes-drift motion fades away, as anticipated above, below (3.7), so that $\psi_{SS}\gg \psi_{SD}$. The trends identified in figure 5 therefore confirm that the Stokes drift can be neglected only if M≫1, whereas for M≲1 it must be necessarily accounted for when seeking an accurate description of the Lagrangian motion, in agreement with previous findings (Raney *et al*. 1954; Chong *et al*. 2013).

To validate the asymptotic prediction $v_{SS}+v_{SD}$, the Lagrangian velocity $v_{L}$ was evaluated from the DNS velocity field for ε=0.1. The value of $v_{L}\left(x,y\right)$ at each location (x,y) was determined by computing the displacement (δx,δy) of a tracer particle, located initially at (x,y), over a cycle (i.e. from t to t+2π), and the resulting velocity $v_{L}\left(x,y\right)=\left(\delta x,\delta y\right)/\left(2\pi \right)$, appropriately rescaled according to $v_{L}/\varepsilon$, was then used to compute the streamlines and vorticity distributions shown in the last column of figure 4, to be compared with the asymptotic predictions shown in the adjacent column. As can be seen, the results are practically indistinguishable, especially for the cases M=1 and M=2, thereby giving additional confidence in the mathematical development. The somewhat larger departures found with M=16, characterized by relative differences in peak streamfunction in the inner and outer vortices of the order of 5%, are to be expected, since, for these values of ε=0.1 and M=16, the relative ordering of the asymptotic development breaks down, in that the viscous term in (2.2) becomes smaller than the convective term. The quantification of these large-Womersley-number configurations can benefit from consideration of the double distinguished limit ε≪1 and M≫1 with $Re_{s}=\varepsilon^{2}M^{2}\sim 1$ proposed in the seminal analyses of Stuart (1963, 1966) and Riley (1965, 1967).

## 4. Fluid-particle drift for finite stroke lengths

The above velocity description, in which the Lagrangian mean motion is the result of the superposition of the steady-streaming and Stokes-drift velocity fields, is rigorously valid only in configurations with small stroke lengths ε≪1, with representative results presented earlier for ε=0.1 in figure 4. There is interest in testing the accuracy with which the asymptotic prediction $v_{SS}+v_{SD}$ describes the fluid-particle drift as the stroke length ε increases to larger values. To that end, we computed numerically the trajectories of fluid particles undergoing multiple oscillatory cycles by integrating

(4.1) $$\frac{\text{d}x_{p}}{\text{ d}t}=\varepsilon v\left(x_{p},t\right),$$

subject to the initial condition $x_{p}=x_{i}$ at $t=t_{i}$, where $x_{p}\left(t\right)$ represents the fluid-particle location scaled with a. The integrations employed the periodic Eulerian velocity v(x,t) obtained in DNS computations of pulsating flows with moderate stroke lengths ε~1. Clearly, for a given initial location $x_{i}$, the resulting trajectory $x_{p}\left(t\right)$ depends on the specific selection of initial time $t=t_{i}$, so that some care must be taken when defining the mean Lagrangian drift when ε is not small, as explained below. For a general discussion of Lagrangian mean flow pertaining to nonlinear waves, the reader is referred to the seminal paper of Andrews & McIntyre (1978).

**Figure 6.**
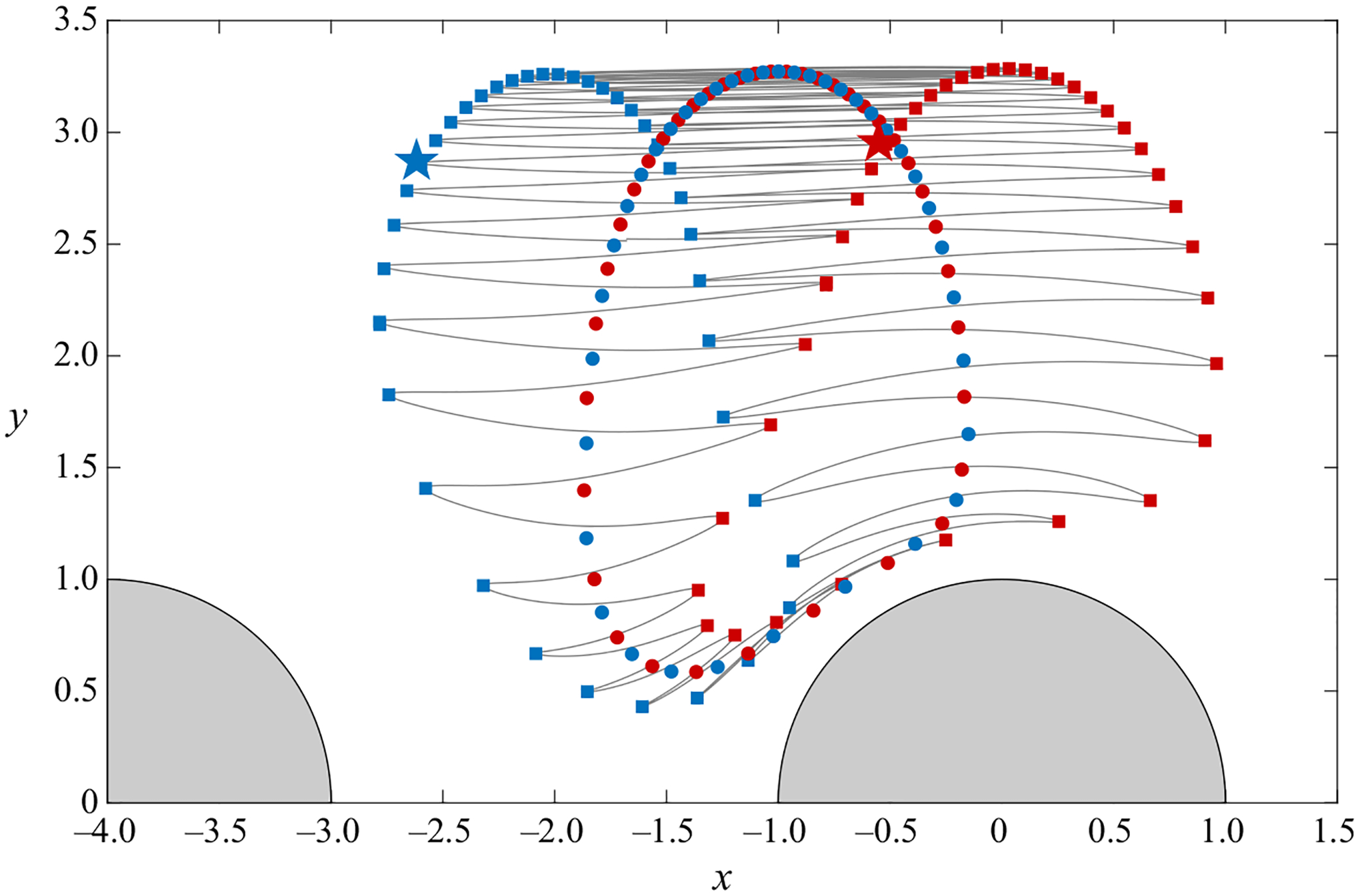
The grey curves represent the oscillatory trajectories determined numerically by integration of (4.1) with initial condition $x_{i}=\left(-0.55,2.95\right)$ (marked with a red star) at $t_{i}=\pi /2$ for M=2, ℓ=2 and ε=1.0. The blue star denotes the particle location at t=3π/2. The squares mark the fluid-particle locations $x_{p}\left(\pi /2+2\pi n\right)$ (red squares) and $x_{p}\left(3\pi /2+2\pi n\right)$ (blue squares) for n=1,2,…, while the circles are the time averages evaluated with use of (4.2) for $t_{i}=\pi /2$ (red circles) and $t_{i}=3\pi /2$ (blue circles).

To illustrate the complications arising in defining the mean Lagrangian drift when ε~1, we plot in figure 6 the results of a representative trajectory calculation, corresponding to oscillatory flow with M=2 and ε=1 about a cylinder array with ℓ=2. Figure 6 shows the path followed by a fluid particle located at $x_{i}=\left(-0.55,2.95\right)$ at $t=t_{i}=\pi /2$, corresponding to the instant of time when the outer velocity $u_{\infty }=\text{cos }t$, decreasing, reaches a zero velocity $u_{\infty }=0$. For illustrative purposes, stars are used to mark the initial location $x_{i}$ (red star) as well as the location x=(−2.62,2.87) (blue star) occupied by the fluid particle at time t=3π/2, when the outer velocity, now increasing from negative values, becomes zero again. The drift motion follows a recirculatory pattern, so that, after a large number of cycles, which would be proportional to $\varepsilon^{-1}$ for ε≪1, the fluid particle returns to occupy a location close to (but not necessarily equal to) the initial location ${\text{x}}_{i}$.

Different options are available regarding the characterization of the Lagrangian drift. One could, for instance, consider the series of locations $x_{n}=x_{p}\left(t_{i}+2\pi n\right)$ occupied by the fluid particle at the end of subsequent cycles n=1,2,…. This series, marked in figure 6 by red squares, serves to delineate the long-time drifting motion of the particle as it circles back towards its initial location following a large number of cycles. One can readily see a problem with this definition, in that, if we had considered instead the fluid particle located at $x_{i}=\left(-2.62,2.87\right)$ (marked by the blue star) at $t_{i}=3\pi /2$, the path followed would be identical, but the Lagrangian drift described by the corresponding sequence of locations $x_{n}=x_{p}\left(t_{i}+2\pi n\right)$, indicated by blue squares, would be radically different, as seen in figure 6.

In trying to characterize the particle drift in a non-ambiguous way, it is therefore better to use instead the average location of the fluid particle during a given cycle n, computed according to

(4.2) $$x_{n}=\frac{1}{2\pi }\int_{t_{i}+2\pi \left(n-1\right)}^{t_{i}+2\pi n}x_{p}\text{ d}t.$$

As can be seen in figure 6, the values of $x_{n}$ corresponding to $x_{i}=\left(-0.55,2.95\right)$ and $t_{i}=\pi /2$, marked by red circles, and those obtained for $x_{i}=\left(-2.62,2.87\right)$ and $t_{i}=3\pi /2$, marked by blue circles, describe the same path, thereby removing the above-mentioned arbitrariness.

As shown in the fourth column of figure 4, for ε≪1 the Lagrangian mean motion features recirculating vortices, whose centre $x_{c}$ can be determined by computing the location where the Lagrangian streamfunction $\psi_{SS}+\psi_{SD}$ shows a local extremum. Similar recirculating patterns are found for ε~1. In that case, the corresponding vortex centre can be obtained numerically by identifying the location $x_{c}$ that satisfies $x_{n}=x_{c}$, so that the fluid particle describes exactly the same trajectory over subsequent cycles, with zero net drift.

**Figure 7.**
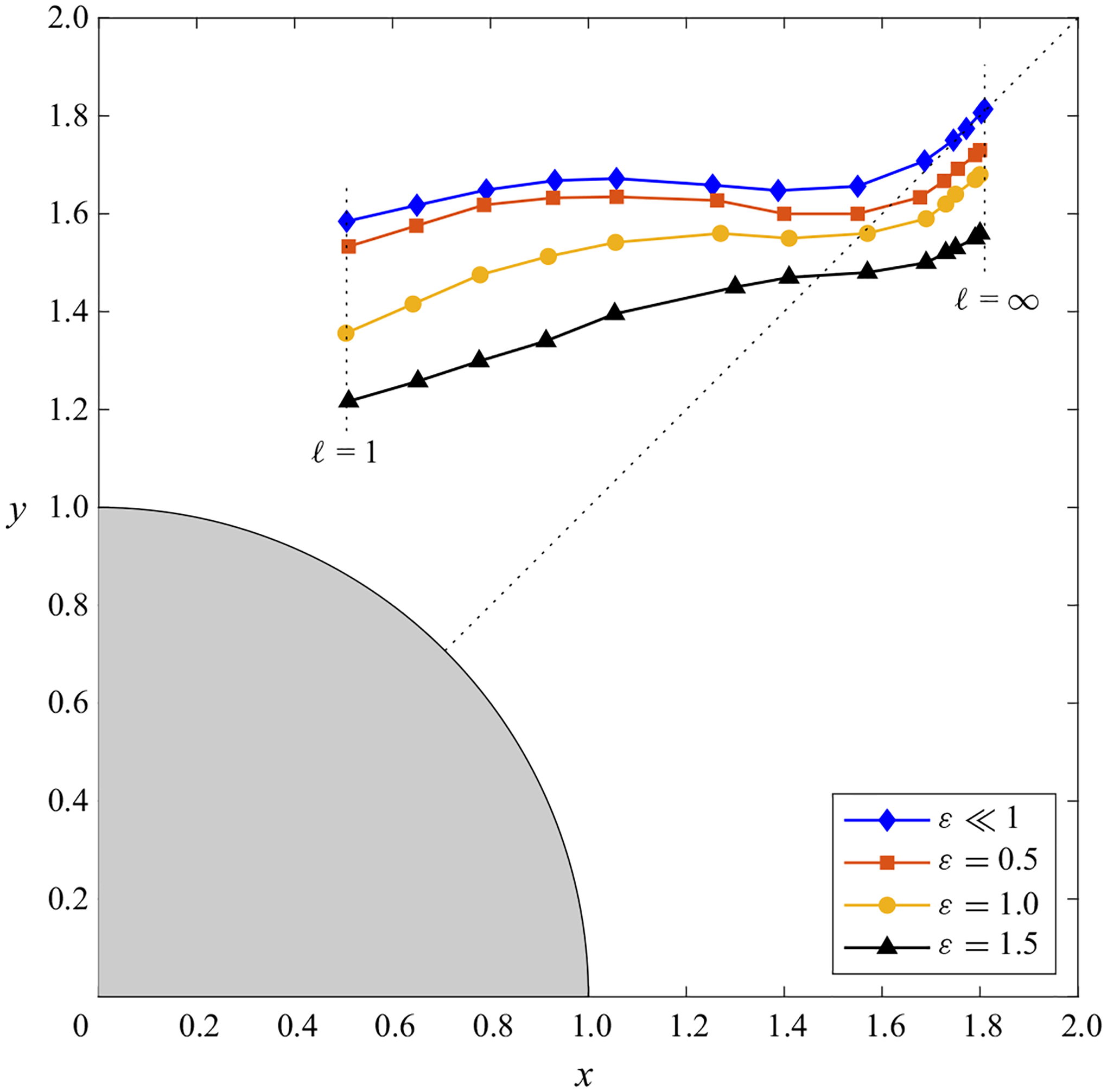
The variation with the inter-cylinder distance ℓ of the location of the Lagrangian vortex centre $x_{c}$ for M=2 as determined in the limit ε≪1 and as determined from the DNS computations with ε=(0.5,1.0,1.5). The symbols represent the results corresponding to ℓ=(1,1.25,1.5,1.75,2,2.5,3,4,6,8,10,15,∞).

The location of the vortex centre $x_{c}$ of the Lagrangian mean flow is shown in figure 7 for oscillatory motion with infinitesimally small values of the stroke length ε≪1 and also with finite values ε=(0.5,1.0,1.5). For the Womersley number M=2 considered in figure 7, there exists a single vortex, whose centre occupies a location that depends on the inter-cylinder spacing ℓ. As can be seen, the results are in general agreement with those displayed in figure 2 for the steady-streaming motion, in that, as ℓ is reduced, the vortex centre migrates from a location near the π/4 ray towards the vertical axis x=0. As expected, the DNS results for increasing stroke lengths ε progressively depart from the ε≪1 predictions, with the vortex centre moving downwards while maintaining approximately the same horizontal location.

**Figure 8.**
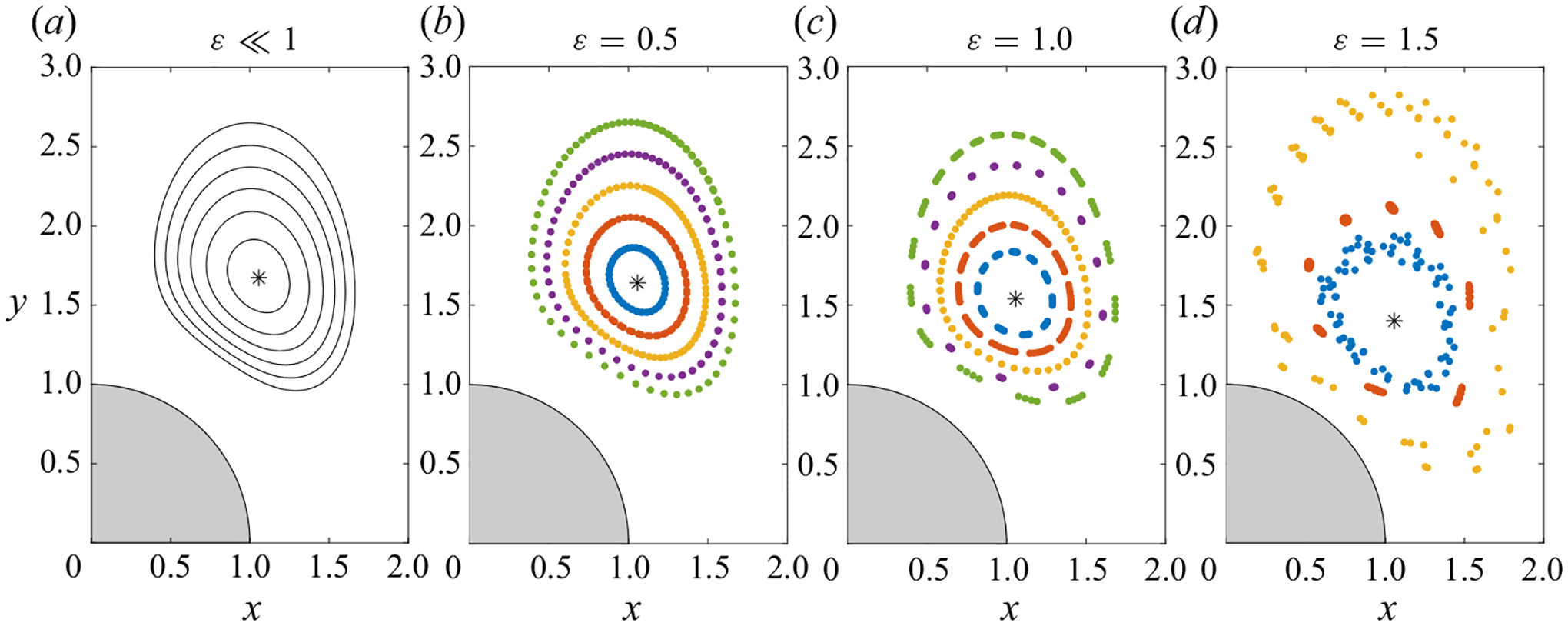
Lagrangian mean motion for ℓ=2 and M=2, including streamlines $\psi_{SS}+\psi_{SD}=$ const. with δψ=0.004 for ε≪1 and time-averaged fluid-particle locations $x_{n}$ for ε=(0.5,1.0.1.5) computed using (4.2) for the trajectories determined by integrating (4.1) with initial condition $x=x_{i}$ at t=0. In computing the trajectories, the initial locations $x_{i}$ were selected at fixed vertical distances δy above the Lagrangian vortex centre $x_{c}$, the latter indicated with an asterisk. Five different trajectories corresponding to δy=(0.2,0.4,0.6,0.8,1.0) are plotted for ε=0.5 and ε=1.0, whereas, to avoid cluttering, only three trajectories corresponding to δy=(0.2,0.6,1.0) are shown in the case ε=1.5.

The increasing downward displacement of the vortex centre for increasing ε shown in figure 7 is accompanied by a progressive distortion of the Lagrangian vortex. This is illustrated in figure 8 for ℓ=2, with the vortex shape characterized by plotting the time-averaged path of fluid-particle trajectories initiated at points located at increasing vertical distances from the vortex centre, indicated in the figure caption. For each fluid particle, the plot shows a sequence of 80 cycles. Since the Lagrangian velocity is larger for larger ε (i.e. $v_{L}\propto \varepsilon$ for ε≪1, as demonstrated in figure 4), for the same number of cycles, the Lagrangian displacement increases with increasing ε, so that, for instance, the fluid particle closer to the vortex centre describes two laps for ε=0.5 and about 10 laps for ε=1.5.

The numerical results for ε=(0.5,1.0,1.5) are to be compared with the Lagrangian streamlines computed in the limit ε≪1 with use of $\psi_{SS}+\psi_{SD}=$ const. As can be seen, the Lagrangian vortex for ε=0.5 is almost indistinguishable from its ε≪1 counterpart and, even for the case ε=1.0, the asymptotic predictions provide a fairly good description of the circular drift motion. Departures are more pronounced for ε=1.5 as a result of the increasing nonlinearity. Contrary to the cases ε=0.5 and ε=1.0, for which all time-averaged locations corresponding to a given fluid particle closely lie along a well-defined closed path, for ε=1.5 the locations $x_{n}$ are scattered within a band surrounding the vortex centre.

The comparisons presented in figures 7 and 8 indicate that the simple velocity description arising for ε≪1, in which the Lagrangian mean velocity is given by the sum of distinct steady-streaming and Stokes-drift components, can be used with unexpectedly good accuracy to quantify the fluid-particle drift in situations in which the stroke length is as large as the cylinder radius (i.e. order-unity values of ε) provided that the flow remains symmetric and periodic. In view of previous results pertaining to the single cylinder (Tatsuno & Bearman 1990), increasing nonlinear effects can be expected to modify significantly the flow pattern depicted in figure 8 as the Reynolds number $Re=\varepsilon M^{2}$ increases to sufficiently large values, with the associated Lagrangian motion eventually becoming chaotic, as the flow becomes turbulent; these additional nonlinear effects were not further investigated here.

## 5. Steady streaming in anharmonically oscillating flows

Most investigations of pulsating flows over cylinders consider outer streams with harmonically varying velocities, resulting in symmetric streaming flows with closed streamlines that are identical in all four quadrants. As shown by Davidson & Riley (1972), the classical analysis can be extended to anharmonic flow by expressing the periodic outer velocity as a Fourier series $u_{\infty }=\int_{n=1}^{\infty }\text{Re}\left(A_{n}{\text{ee}}^{\text{i}t}\right)$ involving the complex coefficients $A_{n}$. Correspondingly, the linear problem that arises at leading order in the limit ε≪1 can be solved by introducing Fourier-series expansions for the velocity $v_{0}=\int_{n=1}^{\infty }\text{Re}\left(A_{n}{\text{e}}^{\text{i}nt}V_{n}\right)$. For the cylinder array, the complex function $V_{n}$ corresponding to a given mode n would be obtained by integrating (3.1a,b) subject to (3.2) for a Womersley number $M_{n}={\left(a^{2}n\omega /v\right)}^{1/2}$. In carrying the analysis to the following order, it is important to note that the forcing term $\langle v_{0}\cdot \nabla v_{0}\rangle$ that determines the steady streaming through (3.3a,b) and the Stokes drift $v_{SD}=\langle \int v_{0}\text{ d}t\cdot \nabla v_{0}\rangle$ are obtained by time averaging the product of two Fourier series. Since the time average of the product of any two modes of different frequency is identically zero, the resulting functions become

(5.1a,b) $$\langle v_{0}\cdot \nabla v_{0}\rangle =\frac{1}{2}\overset{\infty }{\underset{n=1}{\sum }}{\left|A_{n}\right|}^{2}\text{Re}\left(V_{n}\cdot \nabla V_{n}^{\text{*}}\right)\text{ and }v_{SD}=\frac{1}{2}\overset{\infty }{\underset{n=1}{\sum }}\frac{{\left|A_{n}\right|}^{2}}{n}\text{Im}\left(V_{n}\cdot \nabla V_{n}^{\text{*}}\right),$$

involving the sum of the separate contributions of each mode, with no inter-mode interactions. As a consequence, the steady streaming and Stokes drift generated by an anharmonic flow can be obtained simply as the sum of the corresponding steady-streaming and Stokes-drift velocities of each separate mode. Since each mode gives closed streamlines that are identical in all four quadrants, as those represented in figures 2 and 4, their linear superposition also gives symmetrical recirculatory patterns that are qualitatively similar to those obtained in the harmonic case, thereby maintaining the fore-and-aft symmetry of the flow. It can therefore be concluded that the description of the expected symmetry breaking arising in the presence of anharmonic flow requires consideration of the inter-mode interactions occurring at order $\varepsilon^{2}$. These higher-order terms in the asymptotic expansion, which describe the flow asymmetries induced by anharmonic flow, have been computed for circular cylinders and spheres undergoing oscillations with ε≪1 and ${\text{Re}}_{S}=\varepsilon^{2}M^{2}\sim 1$ (Miyagi & Nakahasi 1975; Tatsuno 1981; Higa & Takahashi 1987).

Many oscillatory flow phenomena of physiological interest display an anharmonic time dependence, that being, for example, the case of CSF flow along the spinal canal (Linninger *et al*. 2016). As revealed by magnetic resonance measurements of cardiac-driven motion (Coenen *et al*. 2019; Sincomb *et al*. 2022), the flow rate exhibits a non-sinusoidal variation induced by the intracranial pressure, including a short period of fast caudal flow followed by a longer period of slow flow in the cranial direction. Since this pulsating stream interacts with nerve roots and ligaments that are aligned with the flow, a relevant question is whether such interactions can lead to the appearance of a longitudinal streaming motion, which could explain the enhanced transport rate previously observed (Stockman 2006, 2007).

**Figure 9.**
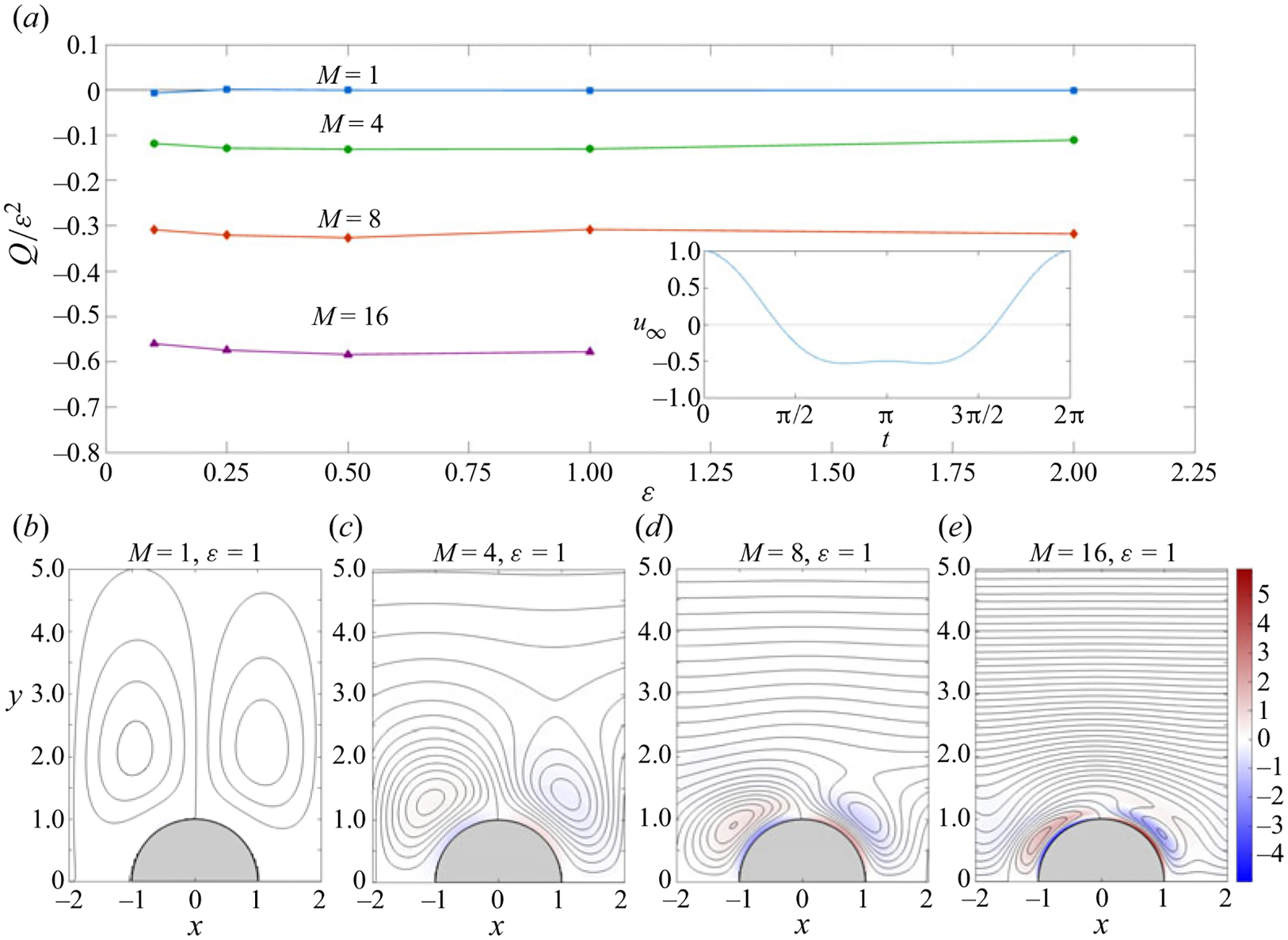
Time-averaged DNS results corresponding to a cylinder array with ℓ=2 and M=(1,4,8,16) for the ambient periodic velocity $u_{\infty }=3\text{ cos}\left(t\right)/4+\text{cos}\left(2t\right)/4$ reesented in the inset in panel (*a*). Panel (*a*) shows the variation with ε of the rescaled streamwise flow rate $Q/\varepsilon^{2}$, while panels (*b*)–(*e*) represent streamlines (with spacing δ〈ψ〉=0.001 for M=1 and δ〈ψ〉=0.006 for M=4,8 and 16) and vorticity contours for ε=1.0.

To try to shed light on this matter, effects of anharmonicity were investigated in connection with pulsating flow over the streamwise cylinder array considered here. In view of the previous comments pertaining to flow over a cylinder, it can be expected that for ε≪1 the velocity corrections associated with the symmetry breaking are small, of order $\varepsilon^{2}$. (Miyagi & Nakahasi 1975; Tatsuno 1981; Higa & Takahashi 1987), so that the appearance of significant asymmetry, possibly leading to a non-zero streamwise flow rate, requires values of the stroke length comparable to the cylinder radius (i.e. ε~1), a problem addressed here with use of DNS simulations. The integrations correspond to a cylinder array with ℓ=2 for a simple two-term periodic ambient velocity $u_{\infty }=3\text{ cos}\left(t\right)/4+\text{cos}\left(2t\right)/4$, whose anharmonic temporal variation is shown in the inset in figure 9(a).

The time-averaged Eulerian velocity 〈v〉 computed for ε=1 was used to determine the streamlines and vorticity shown for four different values of M in figure 9(b–e). The plots include the first two quadrants, as needed to illustrate the lack of fore-and-aft symmetry, which is less pronounced for M=1. For larger values of M, the time-averaged flow comprises two highly distorted vortices in the vicinity of the cylinder, surrounded by a region of nearly horizontal flow with velocities that decay slowly with distance. The comparison of the streaming results for M=1 and M=16 with those shown earlier in the second column of figures 4(a) and 4(c) for the harmonic case clearly indicate that the effects of anharmonicity are much more important for larger values of M, for which the outer vortex is replaced by a streamwise current, which is absent in the case M=1.

The streamline pattern shown in the plots for M≠1 is consistent with the existence of a non-zero streamwise flow rate $Q=\int_{0}^{\infty }\langle u\rangle \left(\ell ,y\right)\text{d}y$ (or $Q=\int_{1}^{\infty }\langle u\rangle \left(0,y\right)\text{d}y$). The variation of Q with ε, determined in the DNS integration from the value of the time-averaged streamfunction 〈ψ〉 in the far field, is shown in figure 9 for different values of M. The plot reveals that the proportionality $Q\propto \varepsilon^{2}$, to be expected for ε≪1, continues to apply over the whole range of ε considered in the DNS, for which the ratio $Q/\varepsilon^{2}$ remains approximately constant. The negative value of $Q/\varepsilon^{2}$, negligibly small for M=1, increases in magnitude for increasing M, reaching $Q/\varepsilon^{2}≃-0.58$ for M=16.

## 6. Concluding remarks

The interaction of an oscillating stream with a streamwise linear array of cylinders gives rise to a stationary motion that has been quantified here for configurations with Womersley numbers M of order unity and dimensionless stroke lengths ε that are either ε≪1 or ε~1, thereby yielding moderately small values of the Reynolds number $Re=\varepsilon M^{2}=U_{\infty }a/v$, for which the flow remains two-dimensional, time-periodic and symmetric with respect to the centreline. For infinitesimally small values of ε, the Lagrangian mean motion is obtained as the sum of the steady-streaming and Stokes-drift components, which have been computed for different values of M and of the inter-cylinder spacing ℓ. The description has been validated by comparisons with results of DNS involving finite values of ε. The comparisons revealed, perhaps unexpectedly, that the simplified description for ε≪1 continues to give reasonably accurate predictions for the time-averaged Eulerian velocity and for the Lagrangian mean motion as the stroke length increases to values of order unity (see, in particular, the results shown for ε=0.5 in figures 3 and 8).

While most of the analysis focuses on oscillating streams with harmonic velocity, consideration is also given to the effects of anharmonicity, an analysis motivated by oscillatory flow phenomena of physiological interest. An important conclusion of our study is that the interaction of an anharmonic stream with a streamwise obstacle array can have a profound effect on the convective transport rate, especially in configurations with ε~1 and large values of M, for which the presence of the array can be expected to induce a streamwise flow rate of order $U_{\infty }a$, corresponding to order-unity values of the dimensionless flow rate Q shown in figure 9.

Further investigation is warranted to assess the importance of these effects in connection with the motion of CSF in the spinal canal, as needed to improve predictive capabilities of current flow and transport models (Sánchez *et al*. 2018; Lawrence *et al*. 2019; Sincomb *et al*. 2022). To enable quantitative predictions, these future investigations should consider more realistic geometrical configurations, including annular models of the spinal canal with obstacles arranged longitudinally to represent the ventral and dorsal nerve roots (Stockman 2006, 2007). The results in §5 suggest that the contribution of the induced Lagrangian motion to the streamwise transport rate is likely to be more prominent in the cervical region, where we find larger values of ε, while associated contributions in the lumbar region will be necessarily more limited.

## Figures and Tables

**Figure 10.**
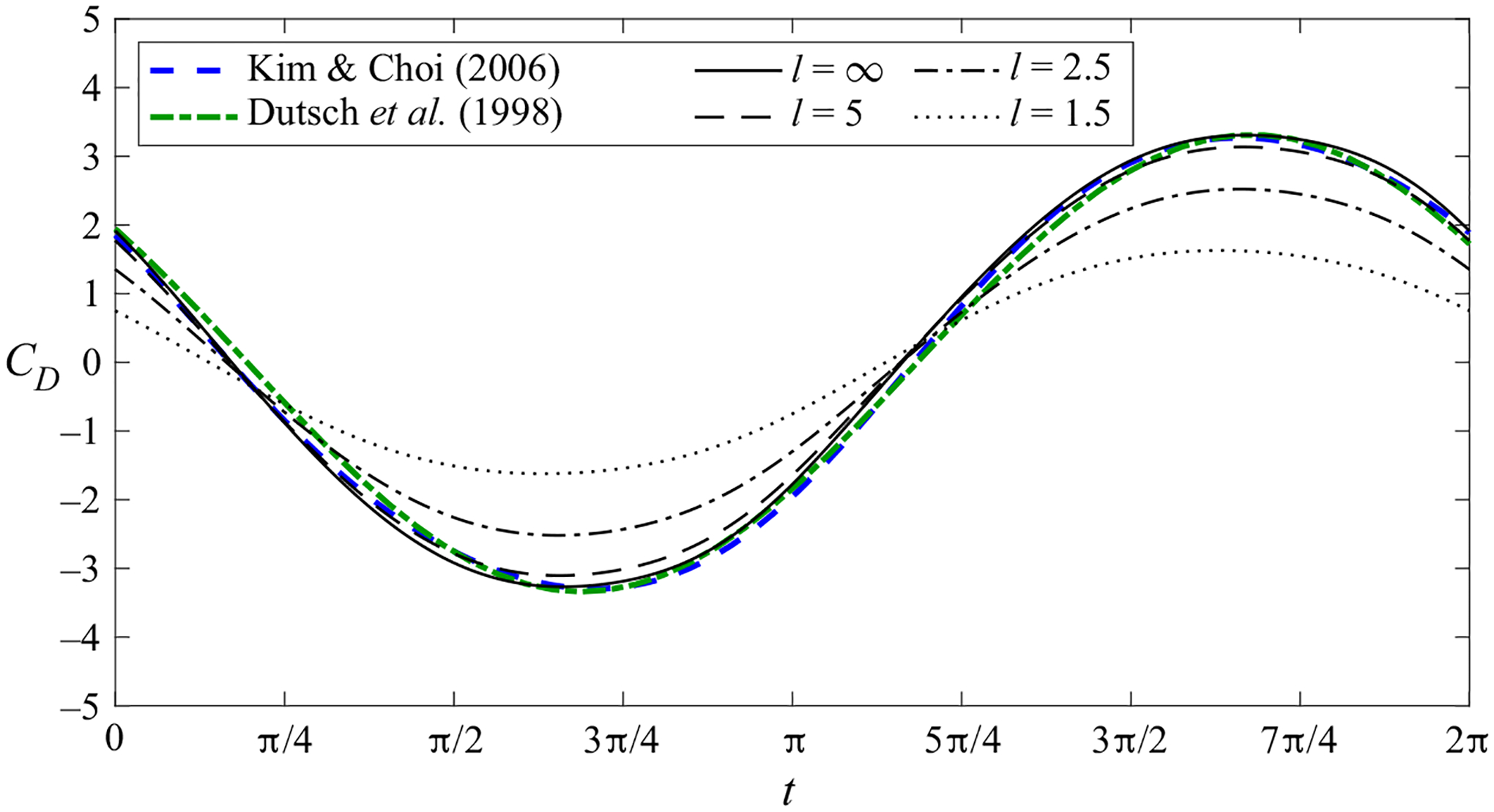
The comparison of the temporal evolution of the cylinder drag coefficient $C_{D}$ for M=5.6 and ε=1.59 reported by Dütsch *et al*. (1998) and Kim & Choi (2006) with results of numerical integrations of (2.1)–(2.5) for ℓ=(1.5,2.5,5,∞).

**Figure 11.**
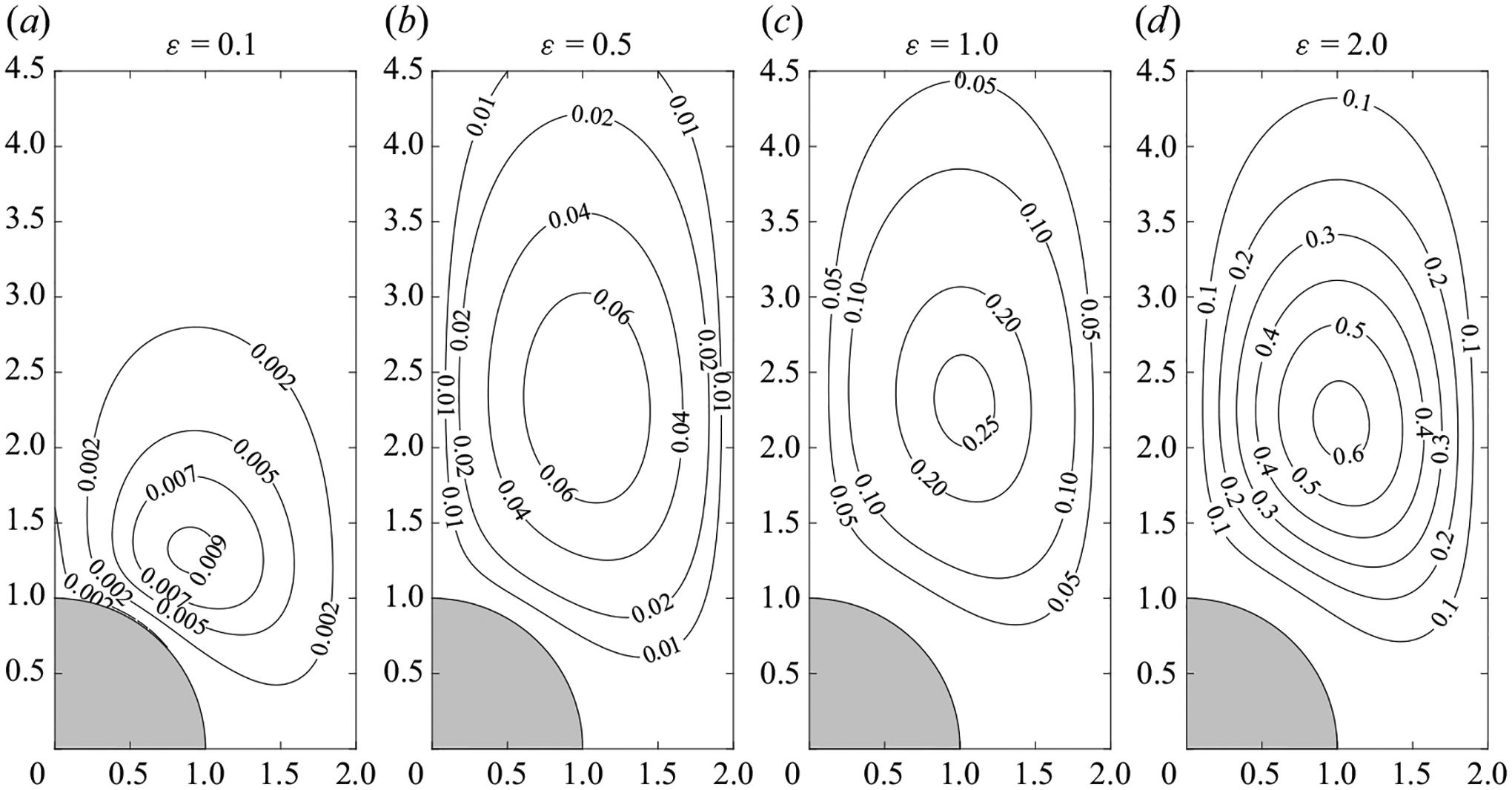
The relative error $\left|\left(\psi_{SS}-\langle \psi \rangle /\varepsilon \right)/\psi_{SS,\text{peak}}\right|$ corresponding to ℓ=2 and M=2 for different values of the stroke length ε.

## Appendix A. Validation of the numerical scheme

The results of the numerical integrations were validated by comparing the temporal variation of the resulting cylinder drag coefficient $C_{D}$ for ℓ→∞ with previous experimental and numerical values reported in the literature for flow over a single cylinder (Dütsch *et al*. 1998; Kim & Choi 2006). As can be seen in figure 10, the resulting curves are virtually indistinguishable. In addition to results corresponding to ℓ→∞, for completeness, figure 10 includes values of $C_{D}$ obtained numerically for different values of ℓ. As expected, the presence of the nearby cylinders reduces the flow velocity in the vicinity of the wall when ℓ≠∞, producing a sheltering effect that reduces the drag as ℓ decreases. For instance, the peak values of $C_{D}$ for ℓ=1.5 are seen in figure 10 to be about half of those of the single cylinder.

## Appendix B. Quantification of error

To facilitate the quantitative comparison between the mean Eulerian velocity determined numerically for finite values of ε and the asymptotic prediction for ε≪1, the results shown in figure 3 are represented in figure 11 using the relative error $\left|\left(\psi_{SS}-\langle \psi \rangle /\varepsilon \right)/\psi_{SS\text{,}\text{peak }}\right|$, where $\psi_{SS\text{,}\text{peak }}=-0.0419$ is the peak value of $\psi_{SS}$. As expected, the relative errors, smaller than 1% for ε=0.1, increase with increasing ε, reaching values exceeding 25% for ε=1.

## Appendix C. Two-time-scale derivation of the Stokes-drift velocity

The familiar expression (3.6) can be systematically derived by considering the displacement of a fluid particle undergoing pulsatile motion with ε≪1, computed from the corresponding trajectory equations

(C1)
$$\frac{\text{d}x_{p}}{\text{ d}t}=\varepsilon v\left(x_{p},t\right),$$

where $x_{p}$ represents the fluid-particle location, scaled with a, and $v=v_{0}+\varepsilon v_{1}+\cdots$ is the Eulerian velocity, which includes a harmonic leading-order term $v_{0}=\text{Re}\left({\text{e}}^{\text{i}t}V\right)$ with zero mean $\langle v_{0}\rangle =0$ and a first-order correction $v_{1}$ having a non-zero steady-streaming component $v_{SS}=\langle v_{1}\rangle$.

The existence of two different time scales in the problem, identified above in the introductory paragraph of § 1, motivates the use of a two-time-scale description in solving Figure 11. The relative error $\left|\left(\psi_{SS}-\langle \psi \rangle /\varepsilon \right)/\psi_{SS\;\text{peak }}\right|$ corresponding to ℓ=2 and M=2 for different values of the stroke length ε. (C1), with the fluid-particle location assumed to be a function ${\text{x}}_{p}\left(t,\tau \right)$ of the two time variables t and $\tau =\varepsilon^{2}t$. Following the classical two-time-scale formalism (Bender & Orszag 1978), we use the chain rule of partial differentiation to write (C1) in the form

(C2)
$$\frac{\partial x_{p}}{\partial t}+\varepsilon^{2}\frac{\partial x_{p}}{\partial \tau }=\varepsilon v\left(x_{p},t\right)$$

and introduce the expansion $x_{p}=x_{0}\left(t,\tau \right)+\varepsilon x_{1}\left(t,\tau \right)+\cdots$, with each term assumed to be 2π-periodic in t. The known Eulerian velocity $v\left(x_{p},t\right)$ appearing on the right-hand side must be correspondingly expanded in the form $v=v_{0}\left(x_{0},t\right)+\varepsilon \left[v_{1}\left(x_{0},t\right)+x_{1}\cdot \nabla v_{0}\left(x_{0},t\right)\right]+\cdots$, leading upon substitution to a hierarchy of problems that can be solved sequentially.

Collecting terms in increasing powers of ε yields at leading order to $\partial x_{0}/\partial t=0$, indicating that $x_{0}={\overset{ˆ}{x}}_{0}\left(\tau \right)$ is a function of only the slow time scale τ. At the following order (ε), one obtains $\partial x_{1}/\partial t=v_{0}\left({\overset{ˆ}{x}}_{0},t\right)$, which can be readily integrated to give

(C3)
$$x_{1}=\int^{t}v_{0}\left({\overset{ˆ}{x}}_{0},\tilde{t}\right)\text{d}\tilde{t}+{\overset{ˆ}{x}}_{1}\left(\tau \right),$$

where $\tilde{t}$ is a dummy integration variable. The Lagrangian mean motion is determined by considering the equation that emerges at order $\varepsilon^{2}$, given by

(C4)
$$\frac{\text{d}{\overset{ˆ}{x}}_{0}}{\text{ d}\tau }+\frac{\partial x_{2}}{\partial t}=v_{1}\left({\overset{ˆ}{x}}_{0},t\right)+\int^{t}v_{0}\left({\overset{ˆ}{x}}_{0},\tilde{t}\right)\text{d}\tilde{t}\cdot \nabla v_{0}\left({\overset{ˆ}{x}}_{0},t\right)+{\hat{x}}_{1}\left(\tau \right)\cdot \nabla v_{0}\left({\hat{x}}_{0},t\right).$$

Taking the time average and accounting for the fact that $x_{2}$ is periodic in t and that $\langle v_{0}\rangle =0$ finally provides

(C5)
$$\frac{\text{d}{\hat{x}}_{0}}{\text{ d}\tau }=\langle v_{1}\rangle \left({\hat{x}}_{0}\right)+\langle \int^{t}v_{0}\left({\hat{x}}_{0},\tilde{t}\right)\text{d}\tilde{t}\cdot \nabla v_{0}\left({\hat{x}}_{0},t\right)\rangle$$

for the Lagrangian mean velocity, which displays the two contributions previously anticipated, namely, the steady-streaming velocity $v_{SS}=\langle v_{1}\rangle$ and the Stokes-drift velocity (3.6).
